# Design and Evaluation of Augmented Reality-Enhanced Robotic System for Epidural Interventions †

**DOI:** 10.3390/s24247959

**Published:** 2024-12-13

**Authors:** Amir Sayadi, Renzo Cecere, Jake Barralet, Liane S. Feldman, Amir Hooshiar

**Affiliations:** Surgical Performance Enhancement and Robotics (SuPER) Centre, Department of Surgery, McGill University, Montreal, QC H3A 0G4, Canada

**Keywords:** haptic feedback, telerobotic, needle insertion, augmented reality, impedance matching

## Abstract

The epidural injection is a medical intervention to inject therapeutics directly into the vicinity of the spinal cord for pain management. Because of its proximity to the spinal cord, imprecise insertion of the needle may result in irreversible damage to the nerves or spinal cord. This study explores enhancing procedural accuracy by integrating a telerobotic system and augmented reality (AR) assistance. Tele-kinesthesia is achieved using a leader–follower integrated system, and stable force feedback is provided using a novel impedance-matching force rendering approach. In this domain, augmented reality employs a magnetic-tracker-based approach for real-time 3D model projection onto the patient’s body, aiming to augment the physician’s visual field and improve needle insertion accuracy. Preliminary results indicate that our AR-enhanced robotic system may reduce the cognitive load and improve the accuracy of ENI, highlighting the promise of AR technologies in complex medical procedures. However, further studies with larger sample sizes and more diverse clinical settings must comprehensively validate these findings. This work lays the groundwork for future research into integrating AR into medical robotics, potentially transforming clinical practices by enhancing procedural safety and efficiency.

## 1. Introduction

### 1.1. Background

Epidural needle insertion (ENI) is a percutaneous clinical technique performed frequently in clinics for a variety of purposes, e.g., pain management and regional/neuraxial blockage. This technique involves inserting and maneuvering a fine, flexible needle through the skin and subcutaneous soft tissue so that the tip of the flexible needle enters the epidural space (ES). The ENI technique is a “blind” minimally invasive procedure [1] in the sense that there is no direct visual feedback from the trajectory of the needle tip through the tissues [2]. Traditionally, once the epidural needle is positioned, the syringe is removed to check for the presence of spinal fluid or blood at the needle hub, indicating potential misplacement into the subarachnoid space or a blood vessel, respectively [3]. This is followed by an aspiration test with a small syringe to ensure that neither cerebrospinal fluid (CSF) nor blood can be aspirated, confirming the needle’s correct placement within the epidural space, which is 3 to 5 cm deep from the skin, with a height of 8 to 10 mm. While ultrasound (US) guidance can enhance needle trajectory visualization, its use in ENI is not common due to the bimanual nature of the procedure and the additional costs involved, making traditional observational and manual confirmation methods the standard practice because of their reliability and simplicity [1,3].

**Figure 1:**
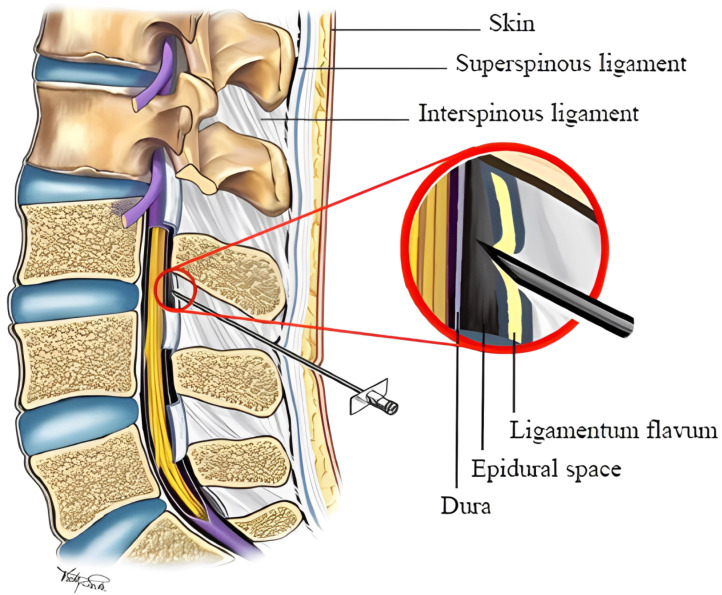
A schematic of the lumbar spine anatomy during an epidural needle insertion procedure [1].

The current standard of care for determining the needle passage through tissue layers and its penetration into the ES is the loss-of-resistance (LOR) technique [4]. For LOR, the physician connects the needle to a syringe filled with saline. The physician then inserts the needle through the soft tissue while pressing the syringe’s plunger. While the needle tip passes through different soft tissues, the physician feels different resistance levels against the saline injection. Eventually, when the needle tip enters the ES, the resistance against the saline injection drastically drops. The physician may then confirm the needle placement in the ES by US. Figure 1 shows a lumbar anatomy and ENI procedure schematic. LOR can also be performed using air. Both air and saline are used to detect the passage of the needle from the denser ligamentum flavum into the relatively less dense epidural space, with the change in resistance felt by the practitioner serving as a critical indicator. While saline provides a more tactile sensation due to its fluid nature, some clinicians favour the air due to factors such as a potentially lower incidence of complications like dural puncture with cerebrospinal fluid leakage, known as a wet tap. The choice between air and saline often depends on the clinician’s training and experience, as well as the specific clinical scenario.

Because of the deep location of the ES, its small size (7 mm wide, 10 mm high, and 5 mm deep), and its proximity to the dura (and spinal cord), the risks of cord puncture and geometrical miss are high in this procedure [1]. Studies have shown that the most common complications of ENI are positional overshoot and accidental puncture of the dura matter (up to 15% [5]). In addition, typically, an epidural injection session takes up to 45 min, most of which is spent on the manual insertion of the needle by trial and error [6].

### 1.2. Alternative State-of-the-Art Approaches

Teleoperated robot-assisted needle insertion (RNI) systems have been successfully adopted for needle insertion into other deep organs, e.g., the prostate [7] and breast nodules [8]. RNI systems have precise and repeatable needle trajectory control advantages and enable physicians to perform teleoperated RNI procedures [8]. Also, studies have shown that RNI systems can perform semi-autonomous needle insertions with a surgeon-in-the-loop control framework. Specific RNI systems for ENI procedures have recently been proposed that adopt similar system architectures [8,9]. Some studies have also proposed custom-designed needles equipped with tip sensors to detect epidural penetration. However, using sensor-equipped needles is not a clinically favourable option due to cost considerations and availability limitations. Also, most of the proposed RNI systems for ENI procedures rely on an imaging modality as a source of intraoperative guidance. The proposed RNI systems typically require preoperative trajectory planning and intraoperative model-based needle control with trajectory error correction.

Clinically, the major limitations of the proposed RNI systems are the following:

1. They rely on preoperative 3D imaging (MRI) for planning;
2. They involve image-based tissue penetration detection, which is prone to error due to large deformations of the soft tissue [8];
3. An imaging device needs to be integrated with the robot controller.

Studies have proposed various designs for needle insertion systems [10]. Robotic systems have been used for needle insertion into soft tissue, e.g., the prostate [7,11,12,13], breast [14,15,16], lung [17], and brain [18]. The proposed robotic platforms in the literature have mainly adopted two design approaches: strap-secured [19] and robotic arm-mounted [20]. While strap-secured needle insertion robots allow superior mobility and a faster setup, they suffer from insertion point instability [19]. Thus, in this study, a robotic arm-mounted design approach was adopted.

Using haptic technology in robotic surgery has become integral in medical procedures, allowing clinicians to interact with digital simulations through tactile feedback. The authors have previously developed medical technologies for force-sensitive interventions, such as laparoscopic, cardiac intraluminal, and percutaneous interventions, e.g., [21,22,23,24,25,26,27,28,29,30,31]. They have also shown that haptic technology enhances surgery precision by enabling the feeling of textures and resistance as if interacting with real tissues, thus improving spatial awareness and decision-making during operations where visual cues are limited [32,33]. These systems provide real-time tactile sensations that significantly enhance procedural accuracy and safety, particularly in scenarios that demand high tactile feedback [34]. Such advancements in haptic feedback not only improve the execution of complex medical tasks but also reduce the likelihood of complications, marking a substantial progression in medical practice.

Recent advancements in medical technology have brought augmented reality (AR) to the forefront, particularly in its application to spinal surgery [35,36]. AR-based methods have shown significant promise in reducing the need for intraoperative X-ray imaging, a standard yet often risky practice in these procedures [37]. The essence of AR navigation lies in its ability to display procedural imagery directly onto wearable devices or screens. This innovative approach allows surgeons to visualize surgical instruments in conjunction with the patient’s anatomy in real time, greatly enhancing precision and safety. Notably, integrating AR with robotic precision has marked a new era in spinal surgery and related procedures. This combination offers unparalleled accuracy, particularly in spinal injections, drawing parallels to the advancements witnessed in other areas of spinal surgery. The precision of robotic systems, known for their ability to execute movements with meticulous accuracy, complements the real-time visual guidance provided by AR. This synergy enhances the surgeon’s ability to perform complex procedures with greater confidence and reduced risk.

Several studies have underscored the efficacy of AR navigation systems, especially when compared to traditional freehand methods [38,39]. Research has consistently shown that the use of AR in spinal procedures, such as the placement of pedicle screws, results in a significantly higher level of accuracy. More importantly, this increase in accuracy is achieved without the need for intraoperative fluoroscopy, a technique that, while useful, exposes patients to radiation. The integration of AR has been linked to a notable decrease in radiation exposure, aligning to minimize patient risk during surgical procedures [36,40].

Furthermore, using AR in spinal surgery enhances procedural accuracy and contributes to overall improvements in surgical outcomes [41]. By providing surgeons with a detailed, three-dimensional view of the surgical site, AR navigation systems facilitate more precise incisions, reduced tissue damage, and potentially quicker patient recovery times. These benefits are of particular importance in spinal surgeries, where precision is paramount to avoiding complications and ensuring successful outcomes [42].

In conclusion, the fusion of AR technology with robotic precision represents a significant leap forward in spinal surgery and related interventions. As these technologies continue to evolve and become more integrated into surgical practices, they hold promise for revolutionizing spinal care, offering safer, more efficient, and highly accurate procedures for patients worldwide [36,40,42].

### 1.3. Objectives and Contributions

To address the limitations of RNI systems for ENI procedures, this study aimed to design, test, and validate a hardware–software integrated needle driver capable of robustly rendering the total needle insertion force to the operator. In addition, it was hypothesized that adapting the authors’ previously proposed impedance-matching method as an alternative to direct force rendering would increase the safety of teleoperation through enhanced stability. More specifically, the contributions of this study are as follows:

1. A novel needle driver system compatible with a commercial medical robot was designed and prototyped, and its performance was validated for ENI tasks;
2. A novel hardware–software integrated system utilizing the proposed needle driver system was developed and integrated with the commercial robot;
3. The authors’ impedance-matching method was extended to be usable for ENI procedures, and its accuracy in force rendering was studied;
4. The integrated system was tested for usability and performance in a multi-user study.

In the following, Section 2 provides a summary of the methodology, Section 4 summarizes the key findings of this study and discusses the results, and Section 5 provides the concluding remarks.

## 2. Methodology

### 2.1. Proposed System Architecture

**Figure 2:**
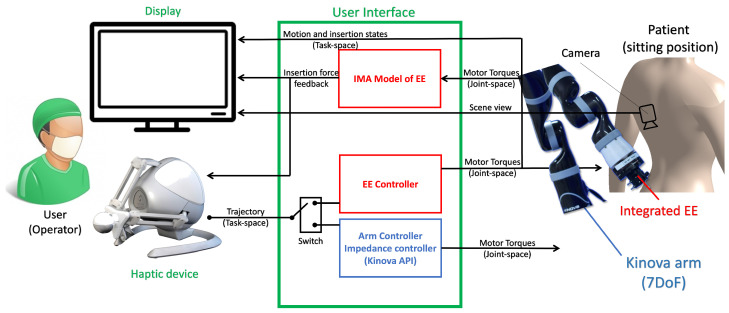
The system architecture of the proposed hardware–software integrated robotic ENI system.

As discussed in Section 1.2, the proposed system was selected to be an arm-mounted system for better stability. Also, leveraging the pre-built control modes of a commercial robot, a 7-degree-of-freedom (DoF) serial manipulator (Kinova Gen2 Spherical, Kinova, Boisbriand, QC, Canada) was used. Figure 2 shows a schematic of the proposed system architecture.

As shown in Figure 2, the system has two operational (control) modes: (a) the robotic arm controller (positioning of the arm) and (b) the end-effector controller (control of needle insertion depth). For the arm positioning mode, two controllers are implemented, i.e., an admittance controller to allow the local aide, e.g., a nurse, to pose the robotic arm in an aiming position on the patient’s skin and a position follower controller for full telepositioning of the arm. A direct insertion force controller (push thrust) was developed for the needle insertion mode. The force controller regulates the thrust on the epidural needle proportionally to the deviation of the haptic device from its zero position. Meanwhile, a force sensor measures the needle insertion force and relays it back to the impedance-matching (IM) force renderer that regulates the force command on the haptic user interface. The system uses a 3-DoF haptic input device (Falcon, Force Dimensions, Nyon, Switzerland) as the operator interface. The haptic device is capable of rendering up to a 15N insertion force. In addition, the serial manipulator is equipped with a 6-DoF force–torque sensor (Gamma, ATI Automation Industries, Apex, NC, USA). The nominal force resolution of the Gamma sensor is 0.01 N.

### 2.2. Needle Driver Design

**Figure 3:**
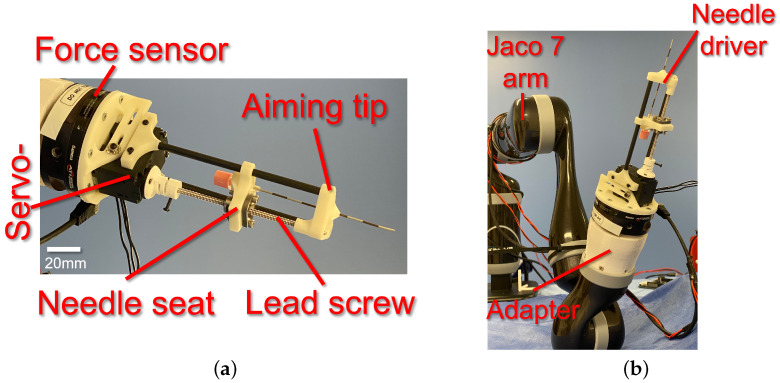
(**a**) Prototyped needle driver end-effector with components and (**b**) integrated needle driver with Kinova Gen2 serial manipulator (Jaco 7 arm) [33].

Figure 3 depicts this study’s prototyped needle driver end-effector. The needle driver provides a total stroke range of 90 mm for an epidural needle with 16–25 G size. The needle driver utilizes a 1 mm pitch lead-screw-and-nut mechanism to drive the needle seat forward and backward (Figure 3a). The lead screw is coupled with a 3D-printed spring coupler to a servo motor (XL-430, Dynamixel, Lake Forest, CA, USA). The servo motor provides an encoder reading of its shaft position with 1024 counts per revolution, resulting in a 1/1024 mm accuracy for the insertion depth measurement. The servo motor is powered and controlled with an OpenCR 1.0 board (Robotis, Seoul, Republic of Korea). An aiming piece (tip) with a central hole was designed and prototyped to provide a line of sight (aiming) to the needle. Also, the aiming tip is connected to the servo motor using two straight fibre-reinforced carbon tubes. In addition, the lead screw serves as the third support, constraining the aiming tip from rotational deviation with respect to the needle driver base.

The needle driver was installed on the Gamma ATI force–torque sensor using a customized 3D-printed adapter. In contrast, the sensor was installed on the manipulator’s end-effector using a customized 3D-printed adapter (Figure 3b).

### 2.3. Cartesian Admittance Controller

A Cartesian admittance controller was developed and implemented to allow the local aide, e.g., the nurse, to position the robotic arm in an aiming position relative to the patient’s spine. The controllers are implemented using C++ object-oriented programming and communicate with the manipulator’s low-level controller (Kinova API in C++), haptic device controller, and force sensor portal using a parallel shared memory protocol.

### 2.4. Arm Telepositioning Controller

For the arm telepositioning controller, first, a Cartesian admittance controller was designed. The manipulator has a native Cartesian velocity controller leveraged to achieve the admittance behaviour. The Gamma force sensor provides real-time external manipulation forces in the end-effector’s coordinate system $f_{ee}$ of the form

(1) $${\tilde{f}}_{ee}=\left(\begin{array}{c} {\tilde{f}}_{{ee}_{x}}\\ {\tilde{f}}_{{ee}_{y}}\\ {\tilde{f}}_{{ee}_{z}} \end{array}\right)$$

Given that the Gamma force sensor is installed on the end-effector, it measures external forces applied on the arm’s end-effector in its local coordinate systems. To map the measured forces to global coordinates, the end-effector’s local-to-global rotation matrix $R_{ee}\in R^{3\times 3}$ is used:

(2) $$f_{ee}=R_{ee}{\tilde{f}}_{ee}$$

In real time, the rotation matrix R is computed every time the end-effector’s Euler angles change (event-based update). The Kinova API provides Euler angles based on a rotated coordinate system with the $x-y^{\prime }-z^{\prime \prime }$ convention, i.e., the Tait–Bryan convention. In the end, a proportional regulation controller was designed for admittance control with the following form:

(3) $$v_{ee}=K_{a}f_{ee}$$

where $K_{a}$ is the diagonal gain matrix. For simplicity, $K_{a}=kI_{3\times 3}$ is adopted, with *k* as the proportional gain selected by the user and $I_{3\times 3}$ as the 3-by-3 identity matrix. The constant *k* also determines the sensitivity of the end-effector to external force. In this study, 0≤k≤1 exhibited stable admittance behaviour and corresponded to a total linear velocity of $0\leq |v_{ee}|\leq 200$$\frac{mm}{s}$.

In addition, the desired Cartesian velocity of the end-effector $v_{ee}$ is augmented by the remote operator’s input on the haptic device. The augmented term to the desired end-effector velocity $v_{ee}^{o}$ is of the form

(4) $$v_{ee}^{o}=k_{h}R_{h}\delta$$

with $\delta ={\left(\begin{array}{ccc} \delta_{x} & \delta_{y} & \delta_{z} \end{array}\right)}^{T}$ being the displacement of the haptic device from its original (resting) position along its x-, y-, and z-axes caused by the remote operator’s hand. Also, the empirical gain $k_{h}=5$ and rotation matrix $R_{h}$ map the haptic device’s coordination system to the end-effector coordination system. Thus, the total desired velocity of the end-effector is

(5) $$v_{ee}^{d}=K_{a}f_{ee}+k_{h}R_{ee}R_{h}\delta$$

where $R_{h}$ is determined empirically as

(6) $$R_{h}=\left(\begin{array}{ccc} 0 & 1 & 0\\ 0 & 0 & 1\\ 1 & 0 & 0 \end{array}\right)$$

Equation (5) shows that the end-effector’s velocity in positioning mode was determined by both the remote operator’s command and the local external forces on the end-effector. If the local external force is exerted due to the collision of the end-effector with an obstacle in the task space, e.g., the patient’s body or the environment, a negative contact force will oppose (override) the remote operator’s command. Thus, this could lead to a safer remote positioning of the end-effector. Also, should the external force be additive to the remote operator’s command (e.g., if a local aide assists in correctly positioning the needle driver), assistive co-manipulation, e.g., for positioning correction or overdrive protection, could be possible.

### 2.5. Needle Insertion Force Controller

**Figure 4:**
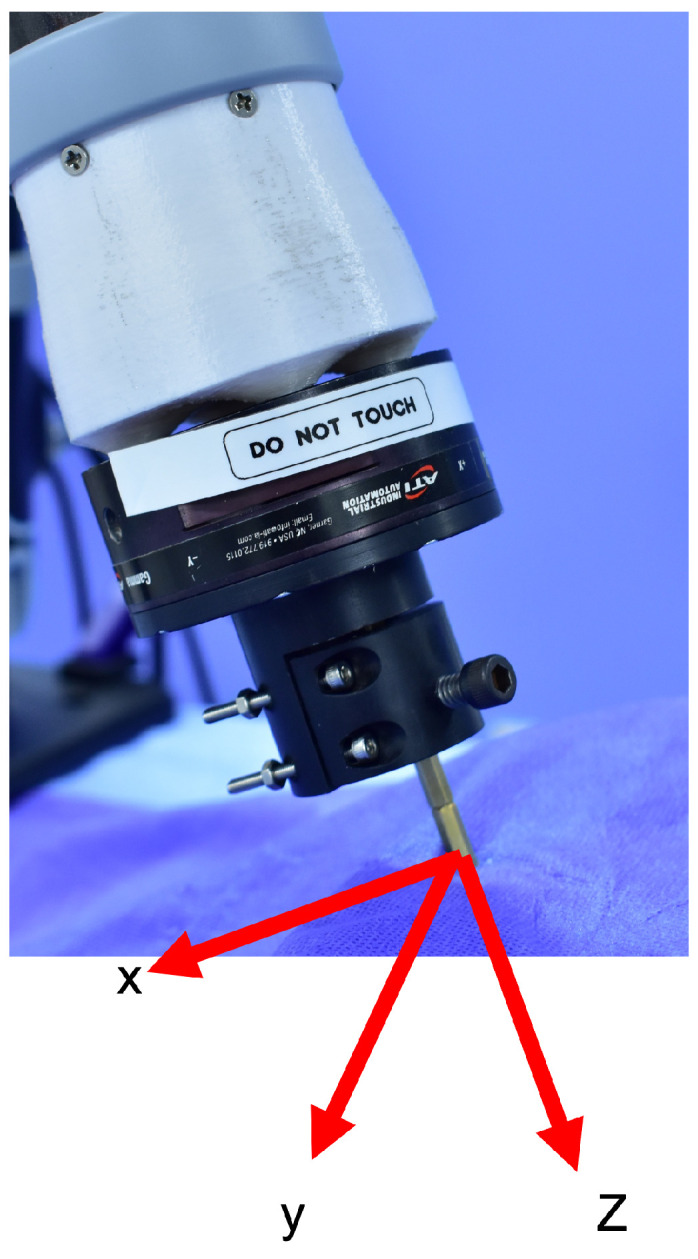
End-effector’s local coordinate system.

After positioning the needle for insertion, assuming that the needle is not extended, it is assumed that upon initial contact with the patient’s skin, the remote operator will switch control to the needle insertion force controller. In this use case, the desired thrust force on the needle along its longitudinal axis (local z-axis, Figure 4) is regulated through the torque control of the servo motor. The utilized servo controller, OpenCR, provides an internal low-level torque controller interface. The desired needle insertion force command is defined as proportional to the displacement of the haptic device from its original position in the local forward axis (local x). Also, given the lead-screw mechanism utilized in the design, a linear relationship between the insertion force and the servo motor’s torque is assumed. Due to negligible friction in the lubricated ball screw (lead screw and lead nut), frictional losses are neglected. Therefore, the desired servo motor torque is defined as

(7) $$\tau =\eta R_{h}\delta \cdot \left(\begin{array}{c} 0\\ 0\\ 1 \end{array}\right)$$

where (·) is the cross-product operator, and η is a heuristic selected by the user on the graphical user interface as the traction coefficient converting the insertion force to servo motor torque. The command τ is calculated in real time and sent to the servo motor driver as the desired set torque.

Meanwhile, to avoid needle breakage if the patient moves perpendicularly to the needle, the end-effector remains under admittance control on its local x-y plane. This combination of force control along the local z-axis and admittance control on the x-y plane also hypothetically increases the safety of remote needle insertion.

### 2.6. IM Force Estimation and Rendering

To render the needle insertion force at the remote operator’s hand, studies have mainly used the direct force reflection (DFR) approach [43,44,45]. In DFR, the end-effector forces are measured using a force sensor or estimated using observers. We have previously shown that, for remote surgical interventions, DFR may suffer from instability caused by a large delay or interruption in communication between the remote haptic device and the robot [46]. If a DFR force rendering were to be adopted in this study, the desired haptic force would be of the form

(8) $$f_{h}=R_{h}^{T}{\tilde{f}}_{ee}$$

Defining *T* as a one-directional time delay between the leader and follower, the error of force feedback would be of the form

(9) $$e_{h}\left(t\right)=R_{h}^{T}\left({\tilde{f}}_{ee}\left(t\right)-{\tilde{f}}_{ee}\left(t-T\right)\right)$$

This would depend on *T* and could potentially surpass the recoverability threshold of the input gradient to the haptic device.

To alleviate this problem, in this study, we adopted the proposed impedance-matching (IM) method of [46] for the robust force rendering of the insertion force. The insertion force feedback at the haptic device is calculated as follows:

(10) $$f_{h}=R_{h}^{T}{\hat{f}}_{ee}$$

where ${\hat{f}}_{ee}$ is the IM-based estimated insertion force, calculated as

(11) $${\hat{f}}_{ee}=K_{IM}{\left(\begin{array}{ccc} \delta & \dot{\delta } & \ddot{\delta } \end{array}\right)}^{T}$$

where $K_{IM}$ is calculated using the method in [46] at the local machine of the remote operator based on received force reading from the Gamma force sensor in the past 50 ms.

As suggested, the passage of the needle tip through layers of soft tissue would hypothetically be identifiable by the user through sudden drops in the insertion force, perceived through haptic feedback [1]. Thus, one of the end-points of the validation studies in this work was to determine the needle tip penetration depth based on pure insertion force feedback in the absence of any imaging modality.

### 2.7. Augmented Reality

**Figure 5:**
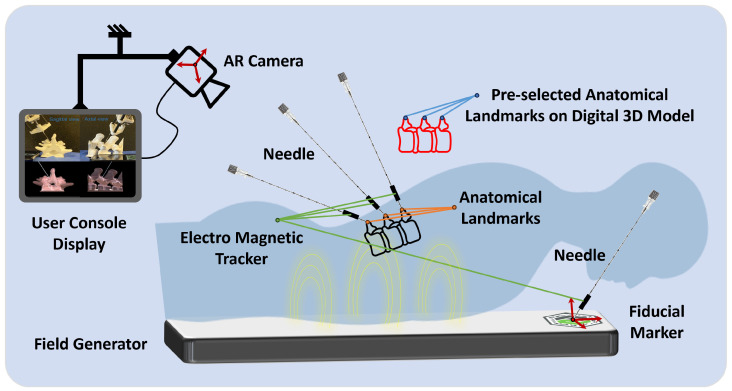
A representative illustration of the registration process using the magnetic tracking system. This diagram demonstrates the calibration and mapping of anatomical landmarks from the patient’s body to the corresponding points on a digital 3D model, enabling a precise augmented reality overlay for surgical planning and guidance.

For the projection of the 3D model onto a 3D-printed counterpart, utilization of a fiducial marker is paramount within the augmented reality framework. This marker is integral to the AR system, providing a reference point for accurately determining the position and orientation of the AR camera (see Figure 5). This alignment is crucial for the projection’s fidelity, enhancing the simulation’s utility and realism in medical training and planning. The calibration framework for the AR system primarily involves using a fiducial marker, specifically a Vuforia tag.

The calibration process begins with a calibration set with a pre-determined relative position of the Vuforia fiducial marker. The position of this marker is ascertained using a 3D measurement system. The primary focus is ensuring the accurate rendering of augmented reality objects in the scene by identifying the AR camera’s pose (position and orientation). Pose estimation is crucial for stable and accurate projections of the 3D-printed model. In this setup, the Vuforia software 10.5 development kit is employed for real-time identification of the camera’s pose concerning the fiducial tag.

The initial step involves generating the anatomical model to create patient-specific 3D holograms for display to the physician. This is achieved by converting a patient’s lumbar CT scan into a detailed 3D digital twin representation using the Mimics Innovation Suite 2021 (Materialise, Belgium). This process allows for the precise and tailored visualization of a patient’s anatomy, significantly enhancing the physician’s ability to plan and execute medical procedures with increased accuracy and confidence.

### 2.8. Digital Twin Registration

The initiation of the augmented reality application within medical procedures is marked by the precise registration of the fiducial marker’s origin, a critical step for determining the AR camera’s spatial location. The marker can be positioned on the patient’s lower back, typically close to the L3–L4 region, to provide a stable reference for AR registration. This placement ensures alignment with anatomical landmarks and falls within the calibrated field of view of the AR camera. This process utilizes a needle with a magnetic tracker (Aurora, Northern Digital Inc., Waterloo, ON Canada) to accurately register the marker’s position. Subsequently, the methodology involves meticulously identifying essential anatomical landmarks in the patient’s anatomy. These landmarks act as pivotal reference points, facilitating the establishment of a direct correspondence between the actual patient anatomy and the digital 3D model derived from medical imaging data. By employing the magnetic tracking system, these selected anatomical landmarks are precisely aligned with their digital counterparts on the 3D model. In this study, three regions of the 3D-printed spinal model (crests of the spinal processes of L4, L5, and L6) were localized using an electromagnetic probe. They were registered to their corresponding regions on the 3D model through static 3D registration. This mapping is achieved through a calibration process where the magnetic tracker precisely locates the predefined points on the patient’s body in the physical space (Figure 5). The system then aligns these points with the digital 3D model’s corresponding landmarks, localized to the fiducial tag coordinate system to overlay the model onto the patient’s anatomy effectively. This alignment is crucial for ensuring that the AR projection accurately represents the patient’s internal structures, providing a real-time, augmented view that enhances the surgeon’s ability to navigate and plan surgical interventions.

## 3. Validation Studies

### 3.1. Setup

**Figure 6:**
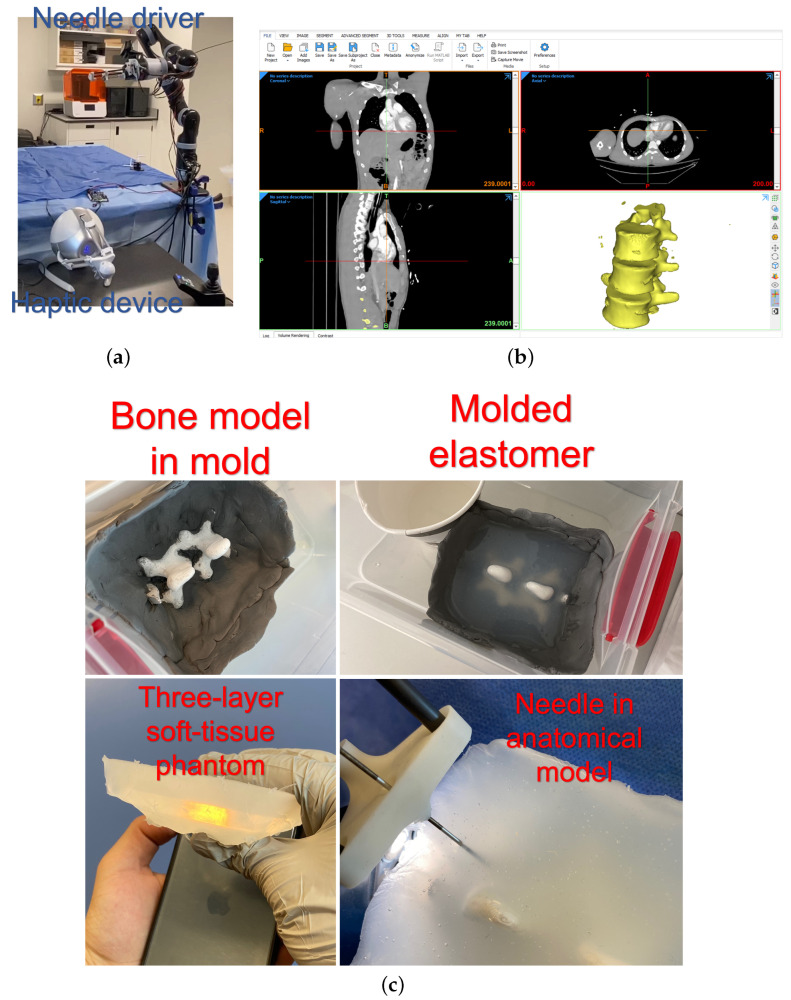
(**a**) The experimental setup, (**b**) CT reconstruction software, and (**c**) the process of moulding the anatomical model with a soft tissue phantom [33].

For validation studies, the setup shown in Figure 6a, comprising the needle driver installed on the serial manipulator and the haptic device, was used. Also, an anatomical 3D model based on a real patient CT scan was fabricated using 3D printing. Three layers of soft-tissue-mimicking material (Ecoflex 00-20, Ecoflex 00-50, and Ecoflex 00-20, respectively) were sequentially moulded on the 3D-printed spinal bone model. Figure 6b,c show the 3D-reconstructed CT scan and fabricated anatomical model. While the AR model is based on preoperative CT images, intraoperative consistency is ensured using a magnetic tracking system. Fiducial markers can serve as landmarks, which allow the device to match real-time information. The allowable limit of such needle path deviations is 1.5 mm. The threshold will be checked continuously, and should the deviation cross this line, the operator will be given an alarm. This technique will be followed for perfect needle trajectories and AR preview accuracy.

### 3.2. Protocol

The validation study protocol was based on the NASA Task Load Index (TLX) to statistically compare the mental demand, physical demand, temporal demand, performance, effort, and frustration in using the robotic system (ROB) versus the robotic system with augmented reality assistance (ROB + AR) versus manual (MAN). Each NASA TLX metric was explicitly defined for participants before the task to ensure consistent interpretation. In addition, the accuracy of force rendering was assessed by evaluating the IM force feedback versus the ground truth (SENSOR). Moreover, the depth of needle penetration into the epidural space was compared between manual and robotic groups.

To perform the analyses, five individuals without prior familiarity with robotic and manual procedures were given 15 min of familiarization with using the robotic system with and without augmented reality assistance and manual procedures before the beginning of the test. During participant tests, both admittance control (x-y plane) and force control (z-axis) were active. This ensured safe and precise needle insertion, simulating realistic clinical conditions. Each individual was tasked to perform five manual, five robotic without AR assistance, and five robotic with AR assistance procedures. The participants were randomly assigned to start with either the manual or the robotic task to mitigate any potential learning or fatigue effects that could bias the results. The participants were asked to stop the intervention when they were convinced that the tip of the needle had penetrated the epidural space.

## 4. Results and Discussion

### 4.1. Needle Penetration Depth

**Figure 7:**
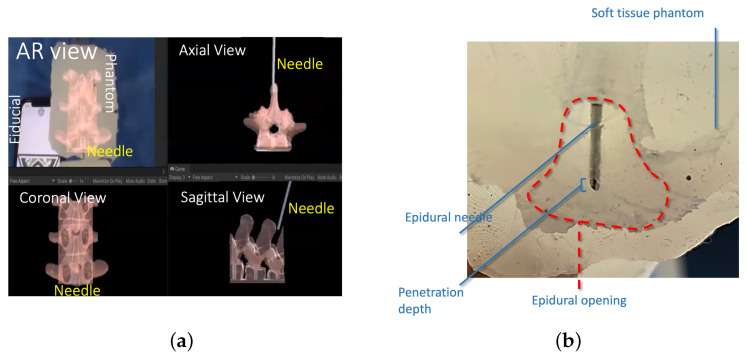

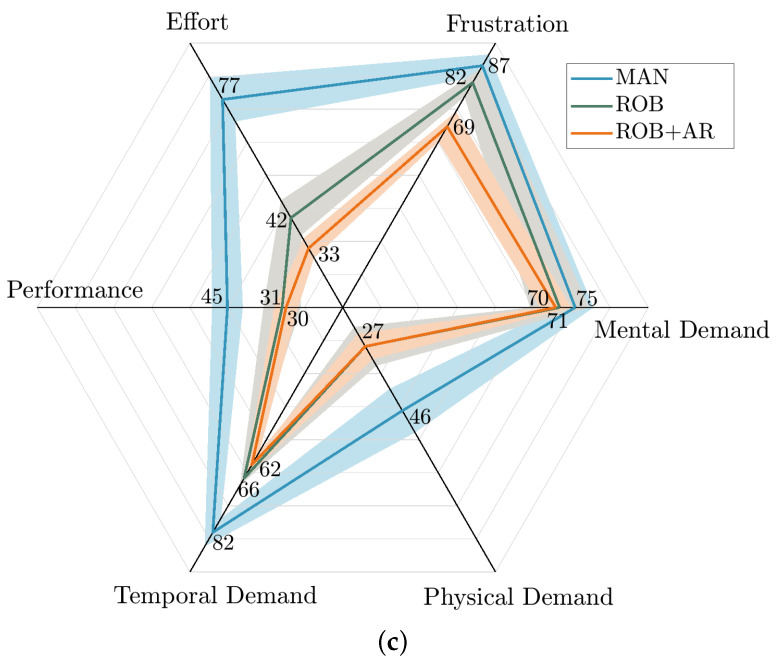
(**a**) A comparison of the sagittal and axial views of the needle in the spine model with a virtual model. (**b**) Penetration depth and epidural cavity representation in a representative post-study image. (**c**) A comparison of TLX results in different procedures [33].

During the procedure, the surgeon observes the augmented model projected onto the patient’s body. Figure 7a illustrates the surgeon console display with sagittal and axial views of the needle in the spine model with a virtual, augmented model. Figure 7b depicts the needle tip after a participant stopped insertion. Table 1 shows the comparative statistics of the needle penetration depth. The results of ROB and MAN groups were statistically tested for statistical differences using an independent t-sample test with a 95% confidence interval (p<0.05 is differently denoted by ★). The t-sample test was chosen for statistical analysis due to its robustness in comparing small sample sizes, commonly used in similar surgical studies. The test ensured the reliable validation of depth and success rate differences between groups. The comparative metrics were time of completion (TOC), needle penetration depth, repeatability (measured by the standard deviation of penetration depth), and success (measured as the percentage of the population successfully inserting the needle into epidural space without retracting the needle). The statistical analysis showed that needle penetration depth, repeatability, and success were statistically different between manual and robotic groups. In all three metrics, ROB (AR) exhibited improved metrics. While traditional methods rely heavily on the practitioner’s expertise and anatomical landmarks, the AR system offers real-time, 3D visual guidance, directly overlaying critical anatomical structures onto the patient’s body. This comparison highlighted an average decrease of 56% in procedural time and an enhanced accuracy rate, suggesting that AR technology not only streamlines the process but also mitigates the risks associated with blind or guided needle insertions.

### 4.2. Force Rendering

**Figure 8:**
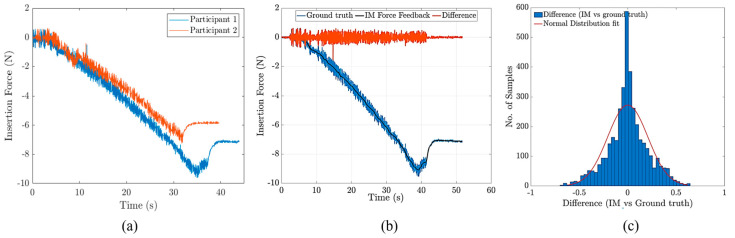
(**a**) Needle insertion forces for two representative participants in the needle insertion study, (**b**) a comparison of the ground truth with IM rendered force, and (**c**) the distribution of the differences between the ground truth and IM force.

Figure 8a depicts the insertion forces recorded for two representative individuals in the study. The experiments were conducted with a sampling frequency of 1 kHz, a needle insertion speed of 2 mm/s, and an 18 G needle diameter to ensure consistency and reliability in the data collection. Two noticeable force drops during needle insertion in all the experiments may have cued the participants to soft tissue layer passages. It was also observed that, in most cases, the second force drop (corresponding to the passage through the last layer and entrance to epidural space) was larger than the first force drop. Also, Figure 8b compares the IM rendered force with the measured insertion force (ground truth) for a representative test. The mean absolute error between the IM force (rendered) and ground truth was 0.23±0.16 N. For the calculation of the mean absolute errors, the first plateau (before insertion) and the forces after the peak (reversal) were not considered to only include the active “insertion” part of the experiments. Figure 8c shows the distribution of accumulated error among all the participants and in all repetitions. The nominal two-way communication delay between the robot and user interface was within the millisecond range. Our empirical testing showed a two-way communication delay of less than 10ms with Ethernet communication.

### 4.3. User Study

After five repetitions of MAN and ROB (with and without AR assistance) had been completed by each participant, they were given the standard NASA TLX questionnaire to indicate their experience with respect to the metrics of the questionnaire (i.e., mental, physical, and temporal demand; performance; effort; and frustration) on a scale of 0 to 100 with increments of 5. The NASA TLX framework uses different full-score indicators for each metric to reflect their relative importance to the overall workload in task-specific contexts. In this study, higher weights were assigned to metrics like performance, frustration, and mental demand, as these are critical for assessing the cognitive load and usability of the robotic and AR-assisted systems. This weighting approach ensures that the Task Load Index accurately represents the specific demands of the procedure. Therefore, given the high-risk nature of the epidural insertion task, the weights in Table 2 were selected to assess the users’ experience. Table 3 shows the results for all five participants.

The results of the TLX analysis showed that the participants’ perceived task load was 18.60 points less with the proposed robotic technology with AR assistance than with manual intervention. The smallest difference in task load metrics was for mental load, with a 5-point improvement with the robotic system with AR assistance, while the largest difference was in effort metric, which showed a 44-point improvement on average among the participants. A contributing factor that might not have been captured in this test is the association among task load metrics, which may alter participants’ perceptions of task loads. Further studies are required to clarify this speculation. Also, the physical demand, performance, and frustration task load metrics were improved in ROB and ROB + AR compared to MAN.

In summary, the findings of the needle penetration depth and TLX analysis showed that the proposed robot-assisted system could improve the accuracy, repeatability, and success rate of needle insertion for the epidural injection tasks. At the same time, it decreased the Task Load Index of this intervention for the participants. Feedback from medical professionals who utilized the AR system during procedures has been decisively positive. Physicians reported an enhanced confidence in needle placement, attributing this to the precise visual cues provided by the AR interface. However, some practitioners noted a learning curve in adapting to the AR visualizations, suggesting the need for targeted training programs to maximize the system’s effectiveness and user comfort. This study was a proof of concept and the first step toward system development for telekinetic epidural needle insertion interventions. Therefore, there were limitations that need to be addressed in subsequent studies. For example, the statistical population of the user study was small. Therefore, the statistical conclusions might be speculative and statistically hard to generalize. Also, the user studies merely focused on the overall system usability, and each module of the proposed system, e.g., the effects of positioning control parameters, the effect of the presence/absence of force feedback, latency in AR visualization, marker recognition in varying surgical environments, and the sampling time window of the IM force rendering system, was not studied. In future studies, using optical tracking systems may increase the accuracy of the registered hologram, potentially leading to sub-millimetre accuracy. Another extension of this study could be to test the usability and performance of the system in other anatomical regions, e.g., kidney stone ablation and deep neural stimulation in the brain.

## 5. Conclusions

In this study, a robot-assisted system for epidural needle insertion was designed, prototyped, controlled, and tested. Performance tests at the system level were performed in a user study with a patient-specific 3D-printed anatomical model. In addition, an augmented reality-guided system was designed and tested for robotic epidural needle injection. Electromagnetic tracker-based static registration was used to register the hologram on a patient-specific 3D-printed model. The preliminary results from our clinical study demonstrate the system’s potential to enhance usability and improve procedure outcomes. Notably, using AR and robotic assistance increased the accuracy, success rate, and repeatability of needle insertions while reducing both the cognitive load on practitioners and the overall procedural time. These promising outcomes suggest that the system could significantly benefit clinical practice by making epidural insertions more efficient and effective. Given these initial findings, there is a clear need for extended research involving larger-scale clinical trials. Such studies will comprehensively evaluate the system’s performance in varied clinical settings, ensuring its reliability and effectiveness in real-world medical applications.

## Figures and Tables

**Table 1:** Statistical comparison of ROB versus MAN groups for needle penetration test.

Group	Needle Penetration Depth Mean (mm)	Repeatability Standard Deviation (mm)	Success (%)	TOC * (s)
MAN	4.3	1.8	94%	18 ± 14
ROB	1.7	1.3	76%	16 ± 4
ROB + AR	1.03	0.77	100%	7 ± 2
Change	−39% ^★^	−40% ^★^	+24% ^★^	−56% ^★^

★: *p* < 0.05; *: time of completion.

**Table 2:** NASA TLX metric weights (0−10).

Metric	Demand			Performance	Effort	Frustration
	Mental	Physical	Temporal			
Weights	7	5	5	10	5	8

**Table 3:** Results of NASA TLX user study survey.

Group	Participant	Demand			Performance	Effort	Frustration	Task Load
		Mental	Physical	Temporal				
MAN	Participant 1	75	30	85	40	90	80	64.75
	Participant 2	60	45	75	55	75	95	67.63
	Participant 3	85	50	75	35	70	90	66.00
	Participant 4	80	65	95	45	90	80	72.50
	Participant 5	75	40	80	50	60	90	66.13
	Mean	75	46	82	45	77	87	67.40
ROB	Participant 1	70	10	65	25	50	95	53.13
	Participant 2	65	35	65	30	25	80	50.50
	Participant 3	90	25	70	45	50	75	60.13
	Participant 4	80	40	60	20	45	85	54.13
	Participant 5	50	25	70	35	40	75	49.38
	Mean	71	27	66	31	42	82	53.45
ROB + AR	Participant 1	75	30	65	25	40	70	50.25
	Participant 2	65	15	60	30	30	80	48.00
	Participant 3	80	35	70	40	25	60	52.25
	Participant 4	70	25	50	25	35	75	47.25
	Participant 5	60	30	65	30	35	60	46.25
	Mean	70	27	62	30	33	69	48.80
	Mean difference (MAN − ROB)	4	19	16	14	35	5	13.95
	Mean difference (MAN − ROB + AR)	5	19	20	15	44	18	18.60
	Mean difference (ROB−ROB + AR)	1	0	4	1	9	13	4.65

## Data Availability

Data are contained within the article.
